# Application of peak frequency annotation after pulmonary vein isolation and its association with long-term atrial fibrillation recurrence

**DOI:** 10.1093/europace/euaf233

**Published:** 2025-09-24

**Authors:** Ming-Jen Kuo, Li-Wei Lo, Jin-Long Huang, Yenn-Jiang Lin, Yu-Cheng Hsieh, Shih-Lin Chang, Yu-Feng Hu, Fa-Po Chung, Cheng-Hung Li, Chin-Yu Lin, Ting-Yung Chang, Ling Kuo, Cheng-I Wu, Chih-Min Liu, Shin-Huei Liu, Yu-Shan Huang, Steven Kim, Shih-Ann Chen

**Affiliations:** Cardiovascular Center, Taichung Veterans General Hospital, Taichung, Taiwan; Institute of Clinical Medicine, National Yang-Ming Chiao-Tung University, No. 155, Section 2, Linong Street, Taipei 112, Taiwan; Institute of Clinical Medicine, National Yang-Ming Chiao-Tung University, No. 155, Section 2, Linong Street, Taipei 112, Taiwan; Cardiovascular Center, Taipei Veterans General Hospital, 201, Sec.2, Shih-Pai Rd, Taipei 112, Taiwan; Cardiovascular Center, Taichung Veterans General Hospital, Taichung, Taiwan; Institute of Clinical Medicine, National Yang-Ming Chiao-Tung University, No. 155, Section 2, Linong Street, Taipei 112, Taiwan; National Chung Hsing University, Taichung, Taiwan; Cardiovascular Center, Taichung Veterans General Hospital, Taichung, Taiwan; Institute of Clinical Medicine, National Yang-Ming Chiao-Tung University, No. 155, Section 2, Linong Street, Taipei 112, Taiwan; Cardiovascular Center, Taipei Veterans General Hospital, 201, Sec.2, Shih-Pai Rd, Taipei 112, Taiwan; Cardiovascular Center, Taichung Veterans General Hospital, Taichung, Taiwan; Institute of Clinical Medicine, National Yang-Ming Chiao-Tung University, No. 155, Section 2, Linong Street, Taipei 112, Taiwan; National Chung Hsing University, Taichung, Taiwan; Institute of Clinical Medicine, National Yang-Ming Chiao-Tung University, No. 155, Section 2, Linong Street, Taipei 112, Taiwan; Cardiovascular Center, Taipei Veterans General Hospital, 201, Sec.2, Shih-Pai Rd, Taipei 112, Taiwan; Institute of Clinical Medicine, National Yang-Ming Chiao-Tung University, No. 155, Section 2, Linong Street, Taipei 112, Taiwan; Cardiovascular Center, Taipei Veterans General Hospital, 201, Sec.2, Shih-Pai Rd, Taipei 112, Taiwan; Institute of Clinical Medicine, National Yang-Ming Chiao-Tung University, No. 155, Section 2, Linong Street, Taipei 112, Taiwan; Cardiovascular Center, Taipei Veterans General Hospital, 201, Sec.2, Shih-Pai Rd, Taipei 112, Taiwan; Cardiovascular Center, Taichung Veterans General Hospital, Taichung, Taiwan; Institute of Clinical Medicine, National Yang-Ming Chiao-Tung University, No. 155, Section 2, Linong Street, Taipei 112, Taiwan; National Chung Hsing University, Taichung, Taiwan; Institute of Clinical Medicine, National Yang-Ming Chiao-Tung University, No. 155, Section 2, Linong Street, Taipei 112, Taiwan; Cardiovascular Center, Taipei Veterans General Hospital, 201, Sec.2, Shih-Pai Rd, Taipei 112, Taiwan; Institute of Clinical Medicine, National Yang-Ming Chiao-Tung University, No. 155, Section 2, Linong Street, Taipei 112, Taiwan; Cardiovascular Center, Taipei Veterans General Hospital, 201, Sec.2, Shih-Pai Rd, Taipei 112, Taiwan; Institute of Clinical Medicine, National Yang-Ming Chiao-Tung University, No. 155, Section 2, Linong Street, Taipei 112, Taiwan; Cardiovascular Center, Taipei Veterans General Hospital, 201, Sec.2, Shih-Pai Rd, Taipei 112, Taiwan; Institute of Clinical Medicine, National Yang-Ming Chiao-Tung University, No. 155, Section 2, Linong Street, Taipei 112, Taiwan; Cardiovascular Center, Taipei Veterans General Hospital, 201, Sec.2, Shih-Pai Rd, Taipei 112, Taiwan; Institute of Clinical Medicine, National Yang-Ming Chiao-Tung University, No. 155, Section 2, Linong Street, Taipei 112, Taiwan; Cardiovascular Center, Taipei Veterans General Hospital, 201, Sec.2, Shih-Pai Rd, Taipei 112, Taiwan; Institute of Clinical Medicine, National Yang-Ming Chiao-Tung University, No. 155, Section 2, Linong Street, Taipei 112, Taiwan; Cardiovascular Center, Taipei Veterans General Hospital, 201, Sec.2, Shih-Pai Rd, Taipei 112, Taiwan; Cardiovascular Center, Taichung Veterans General Hospital, Taichung, Taiwan; Institute of Clinical Medicine, National Yang-Ming Chiao-Tung University, No. 155, Section 2, Linong Street, Taipei 112, Taiwan; Cardiovascular Center, Taipei Veterans General Hospital, 201, Sec.2, Shih-Pai Rd, Taipei 112, Taiwan; Advanced Applications Department, Abbott, Plymouth, MN, USA; Cardiovascular Center, Taichung Veterans General Hospital, Taichung, Taiwan; Institute of Clinical Medicine, National Yang-Ming Chiao-Tung University, No. 155, Section 2, Linong Street, Taipei 112, Taiwan; Cardiovascular Center, Taipei Veterans General Hospital, 201, Sec.2, Shih-Pai Rd, Taipei 112, Taiwan; National Chung Hsing University, Taichung, Taiwan; Division of Cardiology, China Medical University Hospital, Taichung, Taiwan

**Keywords:** Atrial fibrillation, Pulmonary vein isolation, Peak frequency, Bipolar voltage, Conduction gap

## Introduction

Pulmonary vein isolation (PVI) is the cornerstone of catheter ablation for atrial fibrillation (AF), but visual gaps along the ablation line can exist even in the presence of electrical isolation and are associated with recurrence.^1^ While high-density (HD) mapping catheters have improved spatial resolution,^2–4^ conventional voltage mapping fails to identify all conduction gaps.

Our previous work demonstrated that peak frequency (PF) mapping can improve the accuracy of identifying pulmonary vein (PV) conduction gaps. We also established PF cut-off values and showed that PF analysis outperformed conventional voltage mapping by correlating post-ablation remapping signals during the index procedure with conduction gaps confirmed at re-do procedures.^5^ The present study applies these validated thresholds to a larger cohort to assess their prognostic value for predicting long-term AF recurrence, thereby extending PF analysis from validation to clinical outcome relevance.

## Methods

We retrospectively analysed patients with paroxysmal AF who underwent first-time PVI using HD grid mapping catheter and lesion size index-guided high-power ablation at our institution between 1 June 2020, and 31 May 2023. The study complied with the ethical principles of the Declaration of Helsinki and was approved by the institutional review board of Taipei Veterans General Hospital (IRB-TPEVGH number: 2023-08-003CC).

High-density bipolar and omnipolar electroanatomical maps were acquired before and after PVI. Complete PVI was confirmed in all patients during the index procedure by the absence of electrical signals within all PVs, as defined by a voltage threshold of 0.2 mV. Mapping data from both the pre-ablation and post-PVI phases were exported and analysed offline using the Ensite review workstation. On the left atrial (LA) surface, left- and right-sided ipsilateral PV rings were delineated along the encircling ablation lines based on ablation tags and subdivided into eight anatomical segments each, yielding a total of 16 LA-PV sub-regions (see Supplementary material online, *Figure S1*).

For each sub-region, mean bipolar and omnipolar voltage (V_bi_, V_omni_) and PF (PF_bi_, PF_omni_) values were calculated and compared against predefined cut-off values established in our previous study (V_bi_: 0.20 mV; V_omni_: 0.32 mV; PF_bi_: 190 Hz; PF_omni_: 220 Hz). Segments with values exceeding these thresholds were classified as residual electrical activity region. Atrial fibrillation recurrence was defined as any documented episode of AF or atrial tachycardia (AT) lasting longer than 30 s beyond a 3-month blanking period.

Categorical variables were presented as counts and percentages, while continuous variables were expressed as means accompanied by standard deviations. To explore independent associations between clinical variables and AF recurrence, Cox proportional hazards regression analysis was conducted. Sensitivity, specificity, positive predictive value (PPV), and negative predictive value (NPV) were derived from the contingency table. The area under the ROC curve (AUC) was calculated as a measure of diagnostic accuracy. Data analysis was performed using IBM SPSS Statistics (SPSS Inc., Chicago, IL, USA).

## Results

**Figure 1:**
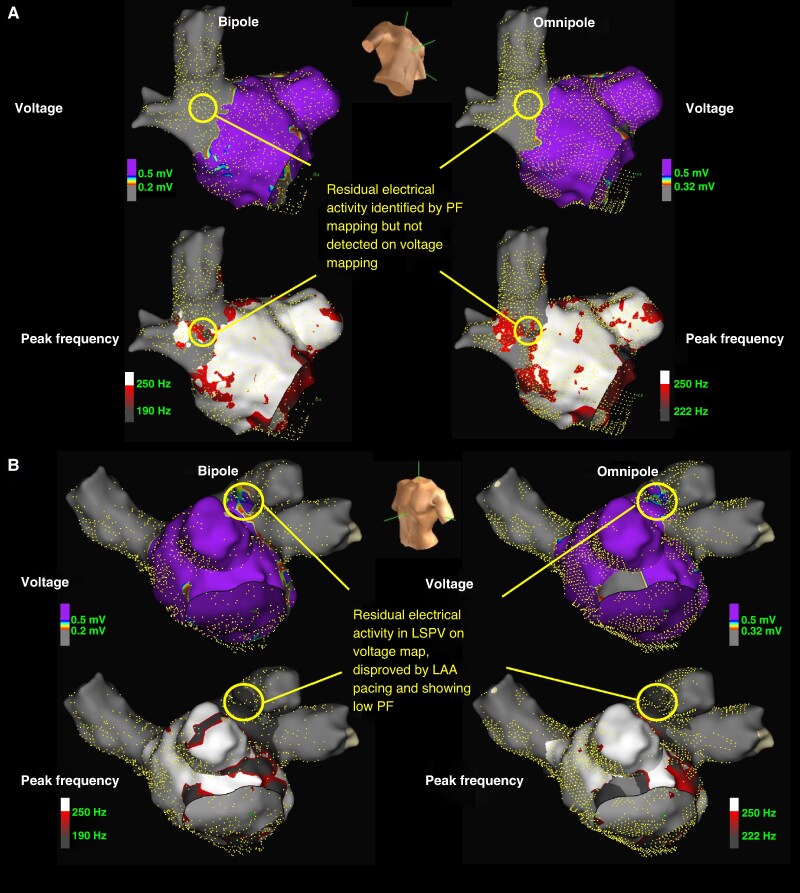
Representative example of applying different criteria over the post-PVI HD map. (*A*) Cut-off values established in our previous study were applied to the post-PVI HD map. Despite low bipolar and omnipolar peak-to-peak voltages over the right anterior carina of the circumferential PV ablation line, high PF signals were still observed in the corresponding region. Additionally, elevated PF persisted in the right carina and right inferior PV, undetected by voltage mapping alone, indicating true conduction gaps identified by PF mapping but missed by voltage mapping. (*B*) High bipolar peak-to-peak voltage was observed over the LAS segment of the circumferential PV ablation line and confirmed as LAA FF potentials via pacing within the LAA. Similar findings were seen using omnipolar voltage criteria. However, both bipolar and omnipolar PF values over the LAS segment and within the left superior PV remained below the defined PF cut-off, indicating the absence of true local activity. Thus, the apparent conduction gap suggested by voltage mapping represented a false positive, as clarified by LAA pacing and PF analysis.

Among 128 patients undergoing first-time PVI, a total of 2048 LA-PV segments were analysed. Based on predefined thresholds, 231 residual electrical activity regions were identified using V_bi_, 178 with V_omni_, 200 with PF_bi_, and 139 with PF_omni_. In the left anterior superior (LAS) segment, 24 of 42 bipolar voltage-defined residual electrical activity regions were confirmed to originate from the LA appendage (LAA) via pacing. Among these, PF_bi_ had a mean of 188.9 ± 57.7 Hz, and only 8 (33.3%) were misclassified as residual electrical activity regions using PF_bi_ criteria. Figure 1 illustrates a representative case.

After adjusting for LAA far-field (FF) signals, the total number of residual electrical activity regions was reduced to 207 V_bi_ in 64 patients, 158 V_omni_ in 58 patients, 190 PF_bi_ in 50 patients, and 132 PF_omni_ in 54 patients. The mean values for V_bi_ and V_omni_ were 0.49 ± 0.43 mV and 0.78 ± 0.57 mV, respectively, while the mean values for PF_bi_ and PF_omni_ were 248.78 ± 57.43 Hz and 275.55 ± 54.98 Hz, respectively.

During a mean follow-up of 21.6 ± 7.4 months, 38 patients (29.7%) experienced AF recurrence—33 with AF and 5 with AT. Recurrences were confirmed by 12-lead electrocardiogram (*n* = 19), 24 h Holter (*n* = 9, mean AF burden 13.2%), or 7-/14-day event recorders (*n* = 10). The presence of residual electrical activity regions, as identified by either voltage or PF criteria, was significantly associated with AF recurrence [numbers of residual electrical activity regions identified in the remapping data, stratified by patients with and without AF recurrence, were as follows: V_bi_: 30 (78.9%) vs. 34 (37.8%); V_omni_: 32 (84.2%) vs. 26 (28.9%); PF_bi_: 36 (94.7%) vs. 14 (16.7%); PF_omni_: 37 (97.4%) vs. 17 (18.9%)]. Multivariable Cox regression confirmed GAP_dormant_—particularly those detected by PF mapping—as the only independent predictor. Moreover, PF-based criteria outperformed voltage-based criteria in diagnostic accuracy for predicting AF recurrence, as reflected by the following performance metrics (sensitivity, specificity, PPV, NPV, AUC): V_bi_: 78.9%, 62.2%, 46.9%, 87.5%, and 0.706; V_omni_: 84.2%, 71.7%, 55.2%, 91.4%, and 0.777; PF_bi_: 94.7%, 83.3%, 70.6%, 97.4%, and 0.890; and PF_omni_: 97.4%, 81.1%, 78.5%, 98.6%, and 0.892. Statistical comparison of ROC curves further confirmed that the AUCs of both PF_bi_ and PF_omni_ were significantly higher than those of V_bi_ and V_omni_ (V_bi_ vs. V_omni_, *P* = 0.0047; V_bi_ vs. PF_bi_, *P* < 0.0001; V_bi_ vs. PF_omni_, *P* < 0.0001; V_omni_ vs. PF_bi_, *P* = 0.0031; V_omni_ vs. PF_omni_, *P* = 0.0020; PF_bi_ vs. PF_omni_, *P* = 0.9054).

## Discussion

This study represents the largest paroxysmal AF cohort (*n* = 128) to date, undergoing HD mapping with both bipolar and omnipolar remapping of 2048 predefined PV segments after index PVI. By applying previously validated PF thresholds, this study highlights the superior utility of PF analysis over traditional voltage mapping in identifying residual electrical activity along the circumferential PV ablation line following PVI in paroxysmal AF.

During cardiac mapping, intracardiac electrogram recordings comprise a mix of near-field (NF) and FF potentials. Accurately distinguishing between NF and FF signals is critical, as mislabelling FF signals may lead to misleading activation maps or unnecessary ablation. Conversely, overlooking small-amplitude NF signals risks underestimating conduction and increases arrhythmia recurrence. Peak frequency–based annotation more accurately differentiated NF from FF potentials, especially in challenging areas like the LAS segment. Unlike bipolar voltage, PF criteria significantly reduced misclassification of LAA FF signals as residual PV activity.

Beyond signal discrimination, PF-defined residual electrical activity regions showed a stronger correlation with AF recurrence than voltage-defined residual activity. Pulmonary vein entrance and exit blocks may not ensure durable isolation, as conduction gaps can persist despite apparent PVI, contributing to PV reconnection and recurrence.^1,6–8^ Even after successful PVI, potentials identified by PF mapping were associated with a higher risk of AF recurrence, offering superior predictive accuracy compared to voltage. Unlike peak-to-peak voltage, PF reflects signal sharpness and local activation, independent of wavefront direction, making it a more reliable indicator of residual conduction.

While EGM sharpness can be assessed visually, this subjective approach introduces operator variability. Peak frequency annotation, by contrast, provides a standardized and intuitive method for identifying residual activity. Integrating PF mapping into routine ablation workflows may improve lesion assessment, reduce unnecessary ablation of FF signals, and enhance long-term rhythm outcomes. Nevertheless, the ultimate proof of clinical benefit would require randomized trials comparing PF-guided additional ablation with conventional mapping approaches. Furthermore, as this study was conducted using radiofrequency ablation, its applicability to the emerging pulse field ablation era remains uncertain. Nonetheless, mapping continues to be important, particularly for differentiating true local activity from FF or LAA signals, and PF analysis may still serve as a valuable adjunct in contemporary practice pending further validation. The recently published EHRA/HRS/APHRS/LHRS expert consensus statement on AF ablation emphasizes the importance of durable PVI and acknowledges the limitations of conventional voltage mapping in detecting residual conduction.^9^ However, PF analysis is not specifically mentioned in the document, reflecting its status as an emerging methodology.

## Conclusion

Peak frequency analysis more accurately identifies residual conduction, which is associated with future AF recurrence after index PVI, suggesting its potential as a reliable adjunct to routine ablation workflows.

## Supplementary Material

euaf233_Supplementary_Data

## Data Availability

The data underlying this article will be shared on reasonable request to the corresponding author.
