# Supplementary figures and images for: Application of peak frequency annotation after pulmonary vein isolation and its association with long-term atrial fibrillation recurrence
Source: Europace. 2025 Sep 24;27(10):euaf233. doi: 10.1093/europace/euaf233 (PMC12510305; doi:10.1093/europace/euaf233)

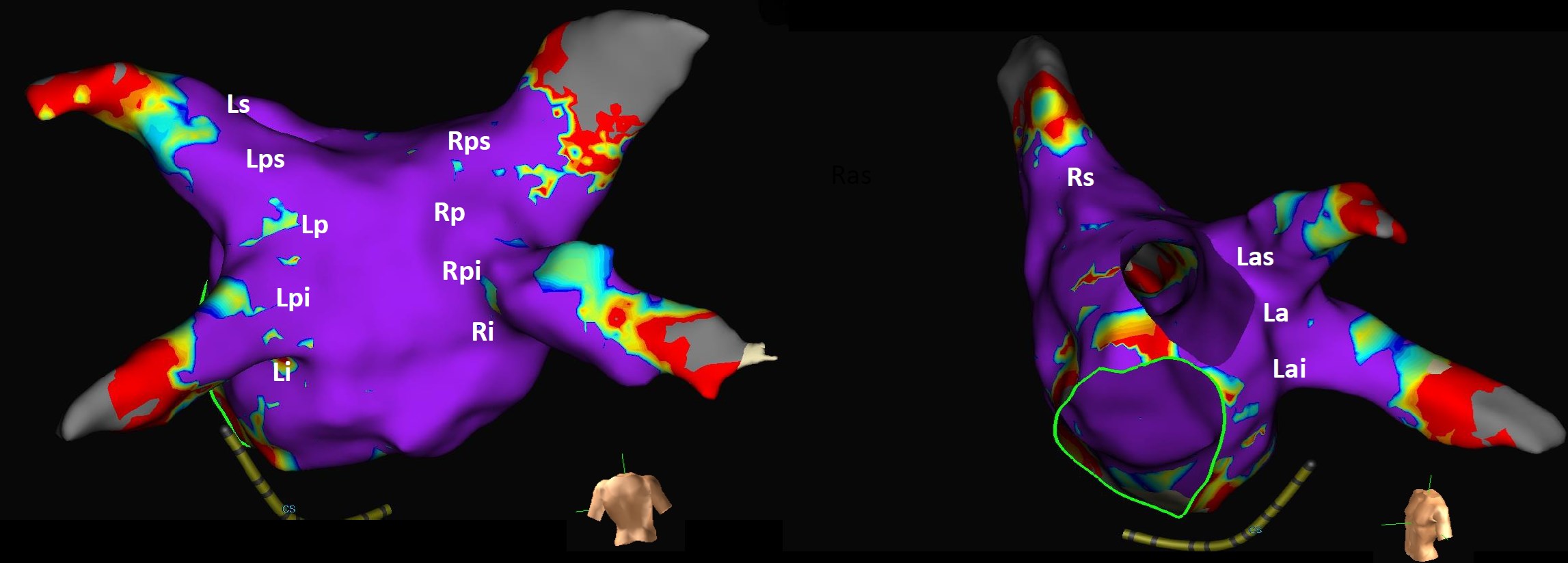

Supplement: euaf233_Supplementary_Data [file euaf233_supplementary_data.jpeg]
